# The *GSTP1* rs1695 Polymorphism Is Associated with Mercury Levels and Neurodevelopmental Delay in Indigenous Munduruku Children from the Brazilian Amazon

**DOI:** 10.3390/toxics12060441

**Published:** 2024-06-19

**Authors:** Mayara Calixto da Silva, Paulo Cesar Basta, Cristina Barroso Hofer, Mirian Akiko Furutani de Oliveira, Joeseph William Kempton, Rogério Adas Ayres de Oliveira, Ana Claudia Santiago de Vasconcellos, Jamila Alessandra Perini

**Affiliations:** 1 Research Laboratory of Pharmaceutical Sciences (LAPESF), State University of Rio de Janeiro (West Zone-UERJ-ZO), Rio de Janeiro 23070-200, RJ, Brazil; 2 Program of Post-Graduation in Public Health and Environment, National School of Public Health (ENSP), Oswald Cruz Foundation (Fiocruz), Rio de Janeiro 21040-900, RJ, Brazil; paulobasta@gmail.com; 3 Department of Endemic Diseases Samuel Pessoa, National School of Public Health (ENSP), Oswald Cruz Foundation (Fiocruz), Rio de Janeiro 21041-210, RJ, Brazil; 4 Department of Infectious Diseases, Federal University of Rio de Janeiro (UFRJ), Rio de Janeiro 21941-630, RJ, Brazil; cbhofer@hucff.ufrj.br; 5 Psychology Division, Central Institute of the Hospital das Clínicas, School of Medicine, University of São Paulo (DIP/ICHC-FMUSP), São Paulo 05403-000, SP, Brazil; mirian.akiko@hc.fm.usp.br; 6 Faculty of Medicine, St Mary’s Hospital, Imperial College London, London W2 1PG, UK; joe.kempton@nhs.net; 7 Faculty of Medicine, University of São Paulo (USP), São Paulo 01246-903, SP, Brazil; roger.adas.doc@gmail.com; 8 Laboratory of Professional Education in Health Surveillance, Polytechnic School of Health Joaquim Venâcio (EPSJV), Oswald Cruz Foundation (Fiocruz), Rio de Janeiro 21040-900, RJ, Brazil; anacsvasconcellos@gmail.com

**Keywords:** mercury exposure, GSTP1, genetic polymorphism, toxicokinetic, neurotoxicity, environmental health, indigenous children, Brazilian Amazon

## Abstract

Genetic polymorphisms may influence mercury (Hg) toxicity. The aims of this study were to evaluate individual factors, such as the presence of the *GSTP1* rs1695 polymorphism, associated with internal Hg dose and child neurodevelopment in indigenous people from the Brazilian Amazon chronically exposed to Hg. Eighty-two indigenous children were clinically evaluated, hair Hg was measured, and the *GSTP1* rs1695 polymorphism was genotyped. The mean age was 4.8 years, the median Hg was 5.5 µg/g, and 93.8% of children exceeded the safe limit (2.0 µg/g). Fish consumption was associated with Hg levels (*p* = 0.03). The *GSTP1* rs1695 *A>G* polymorphism was in the Hardy–Weinberg equilibrium and the highest prevalence of the *GSTP1 AA* genotype (80%) was found in Sawré Aboy, which had the highest Hg levels (10 µg/g) among the studied villages. The Hg levels tended to increase over the years in males and in carriers of the *GSTP1 AA* genotype (0.69 µg/g and 0.86 µg/g, respectively). Nine children failed the neurodevelopmental test, all of whom had Hg > 2.0 µg/g, and 88.9% carried the *GSTP1 AA* or *AG* genotypes, previously associated with the highest internal Hg doses and neurocognitive disorders. The genetic counseling of this population is important to identify the individuals at greater risk for neurodevelopmental disorders resulting from chronic Hg exposure.

## 1. Introduction

In recent years, it has been estimated that artisanal and small-scale gold mining (ASGM), also known as garimpo, has increased by over 90% in the Brazilian Amazon [1]. ASGM is a significant source of human exposure to elemental mercury (Hg^0^), which is carelessly dumped in tons into the environment, allowing its methylation in water bodies and, consequently, its transformation to organic mercury (methylmercury: MeHg), the most dangerous chemical form and a global public health concern [2,3]. MeHg is taken up and accumulated in the aquatic food chain and, after ingestion of fish contaminated with MeHg, can cross the blood–brain barrier and cause damage to the central and peripheral nervous systems, affecting children’s neurodevelopment [4,5,6]. The consumption of Brazil nuts, a very common practice among Amazonian indigenous peoples due to the abundant availability in the region, may interfere with the toxicokinetics of MeHg due to its high concentration of selenium, which has antioxidant properties that allow it to form inert complexes with Hg, thereby reducing the bioavailability of MeHg and toxicity [7].

People who live close to areas invaded by ASGM, mostly Amazonian indigenous peoples, are threatened by its harmful effects and are at risk of developing several health issues [8,9,10]. By causing neuropsychiatric damage, Hg exposure resulting from this activity has a direct impact on people’s quality of life [11,12,13,14,15,16,17]. A recent study showed a tendency of worse mental health indicators in Munduruku indigenous people with elevated levels (≥6.0 µg/g) of MeHg exposure [11]. Development is a dynamic process that permeates all stages of life and can be influenced by hereditary characteristics and experiences offered by the environment [18]. Risk factors for developmental delays include iron deficiency anemia, exposure to environmental pollutants, malaria infection, stunting, maternal depression, inadequate stimulation and learning opportunities, exposure to violence [19,20,21], and genetic factors [22].

It is known that genetic polymorphisms can influence the internal Hg dose [14,15,23] and, consequently, its signs and symptoms of exposure [14,15,24,25]. In our previous study, we observed the association of the *GSTP1* rs1695 single-nucleotide polymorphism (SNP) with Hg levels and neurological signs in indigenous adults of the Munduruku ethnic group from the Brazilian Amazon [15]. MeHg is mainly metabolized through its conjugation to small tripeptide glutathione (GSH), catalyzed by the glutathione S-transferases, particularly the pi 1 isoform (GSTP1), that allows its elimination via the ABC transporter system in the bile [26,27].

Therefore, the aims of this study were: (1) to evaluate the associations between demographic and clinical characteristics, fish and Brazilian nut consumption, and internal mercury dose; (2) the interaction of the *GSTP1 313 A>G* (rs1695) polymorphism on the internal mercury dose; and (3) the association between the internal mercury dose and the Denver II test results to identify Munduruku indigenous children chronically exposed to MeHg who are, therefore, eligible as a priority for health intervention applications. 

## 2. Materials and Methods

### 2.1. Study Design and Population

This study was part of a major project approved by the National Ethics Committee of Human Research (protocol number 65671517.1.0000.5240) and described by Basta et al., 2021 [8]. A cross-sectional study was carried out between October and November 2019 with residents from three villages (Poxo Muybu, Sawré Aboy, and Sawré Muybu) of the Munduruku people of the middle Tapajós River in Pará state, Brazilian Amazon.

**Figure 1:**
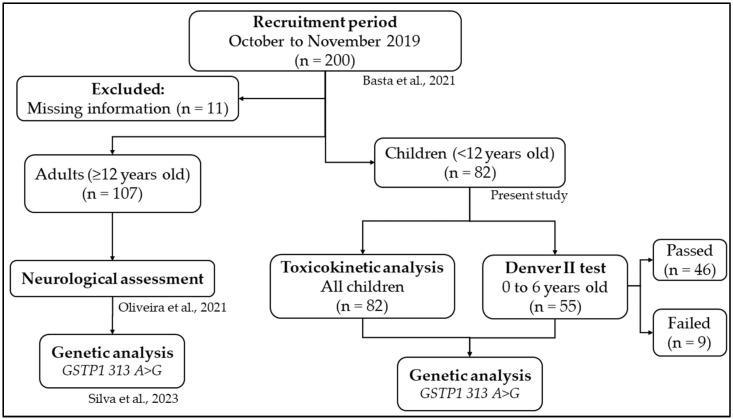
Strategy for the analysis of the associations between the *GSTP1 313 A>G* polymorphism, Hg levels, and neurodevelopmental outcomes in the Munduruku people of the middle Tapajós River in Pará state, Brazilian Amazon. Basta et al., 2021 [8], Oliveira et al., 2021 [16], Silva et al., 2023 [15].

All families were invited to participate in the study, and there were no refusals. The major project achieved a sample of 200 individuals, and for the present study, a convenience sample of 82 children from 0 to 11 years old was selected without any probabilistic sampling methods. The associations between the GSTP1 313 A>G (rs1695) polymorphism, Hg levels, and neurological conditions were already evaluated in individuals 12 years or older [15] (Figure 1).

**Figure 2:**
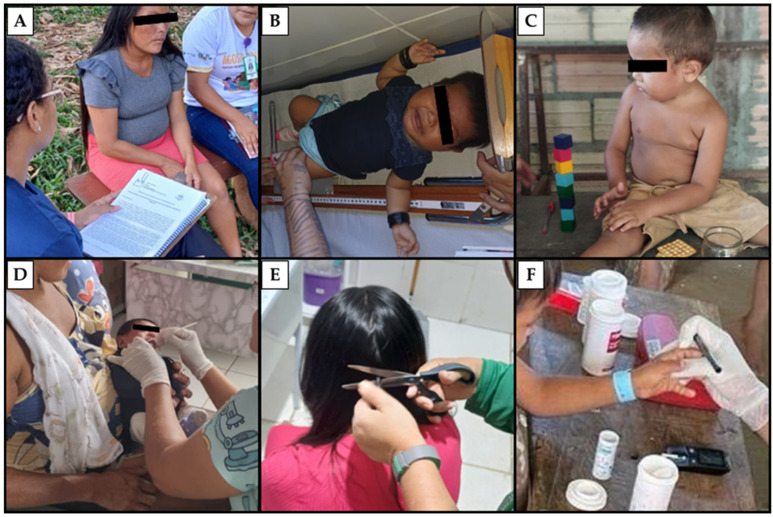
Photos of the study to illustrate the overview of the fieldwork steps, data, and biological sample collection. (**A**) Application of the sociodemographic questionnaire and ICF. (**B**) Children’s clinical and pediatric evaluation. (**C**) Application of the Denver II neurodevelopmental screening test. (**D**) Oral mucosal epithelial cells were collected with a sterile swab for DNA extraction and polymorphism analysis. (**E**) Hair samples were taken near the scalp in the occipital region to determine mercury levels. (**F**) To measure capillary hemoglobin, we collected a drop of blood from the finger using a sterile lancet.

After obtaining written consent via the informed consent form (ICF) from the children’s guardians, an initial interview was conducted to gather sociodemographic data, as well as fish and Brazilian nut consumption. Subsequently, all participants underwent clinical evaluations and provided biological samples, as illustrated in Figure 2.

### 2.2. Clinical and Pediatric Evaluation

Initially, the anthropometric measurements and determination of hemoglobin were performed as previously described [8]. The Z-scores (adjusted for sex and age) of the height for age (HA), weight for age (WA), weight for height (WH), and BMI for age (BA) were calculated according to the WHO reference population [28] and the Z-score values were categorized according to guidelines from the Brazilian Ministry of Health as follows: severely stunted, moderately stunted, and normal (HA), severely underweight, moderately underweight, normal, and overweight (WA), and at risk of eutrophication and overweight (WH and BA) [29]. Following the WHO guidance on anemia, children aged 6 to 24 months with <11.0 g/dL of hemoglobin (Hb) and children aged 2 to 6 years with <11.5 g/dL of hemoglobin were classed as anemic [8,30]. 

All children underwent a clinical evaluation performed by an experienced pediatrician, in which child vaccination registration booklets and booklets for prenatal care were assessed, and the Denver II neurodevelopmental screening test was applied. The Denver II test has been used in several countries to monitor the development of children from 0 to 6 years of age who have associated risk factors and is composed of 125 items to evaluate children’s development in four areas: (i) personal and social; (ii) fine motor skills; (iii) language; and (iv) gross motor skills. The items are organized in increasing order of difficulty and applied directly to the child or asked by the caregivers. The items corresponding to the child’s age are conducted, as well as some items from the previous and subsequent ages, and then, each one is evaluated according to the child’s performance. A child is considered “advanced” when they pass an item that succeeds their chronological age, “normal” if all the items of the corresponding age are completed, and “delayed” if only the items below their chronological age are completed. Therefore, to carry out the analyses for the present study, the “advanced” and “normal” results were classified as “passed”, and the “delayed” results were classified as “failed” [31,32].

### 2.3. Hair Mercury Analysis

Participants’ internal total Hg dose (exposure levels) was estimated from the hair samples collected from the occipital region of the scalp using stainless steel dissection scissors. The samples were then stored in individually identified paper envelopes. Afterward, the samples were sent to the Toxicology Laboratory in the Environment Section of the Evandro Chagas Institute (IEC) in Belém-Pará, Brazil, for the analysis of the total mercury levels (THg). The detailed methodology was described by Basta et al., 2021 [8]. The THg from hair samples is known to be a reliable biomarker of MeHg exposure through the consumption of contaminated fish since MeHg is the predominant form of mercury found in the hair THg [33].

Following the WHO recommendations, an internal dose of mercury <2.0 µg/g in hair was considered as the safe limit and, therefore, Hg was categorized as <2.0 µg/g or ≥2.0 µg/g [34]. Additionally, the analyses were conducted using the median hair Hg level of the overall studied population (n = 82) as a cutoff point.

### 2.4. DNA Extraction and GSTP1 Genotyping

To access the DNA of the participants, epithelial cells were collected from the oral mucosa using sterile swabs. Then, the samples were stored in an individually identified microtube containing a buffered solution and transported to the Laboratory of Pharmaceutical Science (LAPESF) of the State University of Rio de Janeiro, West Zone Campus, in Rio de Janeiro (RJ). The genomic DNA extraction from the samples was performed with an extraction kit (Qiagen, Hilden, Germany) following the manufacturer’s instructions. Briefly, the samples were incubated at 56 °C with 20 µL of proteinase and 400 µL of lysis buffer to release the intracellular material. The mixture was then centrifuged, and 400 µL of ethanol was added, allowing DNA precipitation. Then, 700 µL of the mixture was transferred to a silica column, which, after centrifugation, retained the DNA. Finally, two washing steps and an elution step of the purified DNA were performed.

Finally, the TaqMan allelic discrimination assay (C_3237198_20) with the 7500 Real-Time System (Applied Biosystems, Foster City, CA, USA) was performed for the genotyping analysis of the *GSTP1* (chr11:67585218) *313 A>G* (rs1695) missense variant, as previously described [14], and the *GSTP1 313 A>G* allele frequency and genotype distribution were derived via gene counting, as shown in Figure 2. 

### 2.5. Data Analysis

The continuous variables were presented as the mean, median, and their respective range, with linearity tested with the Shapiro–Wilk normality test. An analysis of variance (ANOVA) and Tukey’s honest significant difference (HSD) test were used to investigate the differences between the villages. The correlations between the continuous variables were tested with Spearman’s linear correlation coefficient and linear regression models with possible effect-modifying factors. 

The categorical data were presented as the number (n) and frequency (%), and the differences between the groups were calculated using the Chi-square test or Fisher’s exact test, if necessary. The Hardy–Weinberg equilibrium (HWE) for the *GSTP1 313 A>G* polymorphism was calculated using the goodness-of-fit test. All statistical analyses were performed using R Software (R Foundation for Statistical Computing, Vienna, Austria, version 4.2.2), and a significance level of 5% was adopted. 

## 3. Results

The study population was composed of 82 children from 0 to 11 years of age residing in the Poxo Muybu (34.1%), Sawré Aboy (18.3%), and Sawré Muybu (47.6%) villages from the Sawré Muybu indigenous territory in Pará State, Brazilian Amazon. In the overall population, most individuals were female (51.2%), and the median age was 4.0 years old, which was used to categorize this continuous variable. However, no significant differences were observed between the villages. Z-scores of HA, WA, and WH, as well as the prevalence of anemia and the Denver II test, were also not statistically different between the villages, even considering the original continuous variables, when applicable. Regarding fish consumption, most residents (82.1%) from the Sawré Muybu village consumed fish at least three times a week (*p*-value = 0.008) compared to Poxo Muybu and Sawré Aboy (39.3% and 66.7%, respectively), and Brazilian nut consumption was similar between the villages. In addition, 16.4% of the children failed at least one domain of the Denver II test. However, there was no significant difference between the villages. Table 1 shows the sociodemographic and clinical data of the study population, comparing the three villages analyzed.

**Figure 3:**
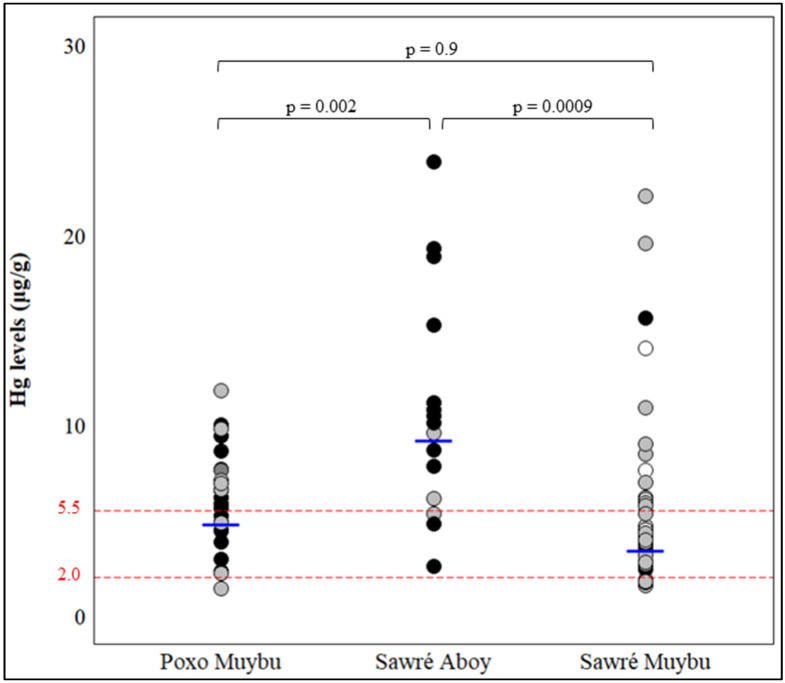
Distribution of hair Hg levels by village. The *p*-value for the pairwise comparison of villages obtained with Tukey’s HSD test. The red lines indicate the safety limit (2.0 µg/g) and the population median (5.5 µg/g), while the blue dashes indicate the median for each village. Each circle corresponds to the level of Hg in the hair of each individual. The circles are colored according to the *GSTP1* rs1695 *A>G* genotypes: (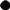) black for *GSTP1 AA*, (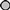) gray for *GSTP1 AG*, and (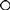) white for *GSTP1 GG*.

**Figure 4:**
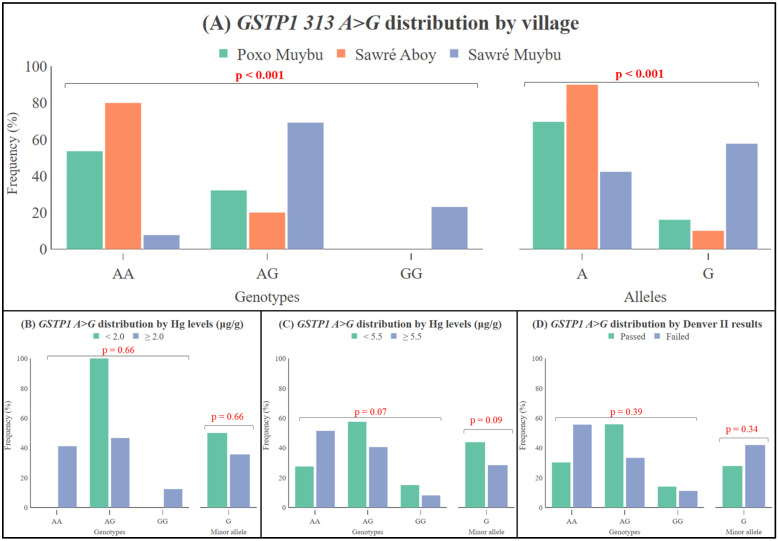
Distribution of the *GSTP1 313 A>G* (rs1695) genotypes (*AA*, *AG*, and *GG*) and alleles (*A* and *G*) according to the villages (**A**) according to the Hg internal dose (**B**,**C**) and the Denver II test results (**D**). The *p*-values obtained from the Chi-squared test (Pearson’s *p*-value).

The hair Hg levels did not show a normal distribution (*p*-value < 0.05, Shapiro–Wilk normality test), and the median from the overall population was 5.5 µg/g. The median Hg levels differed between the villages (ANOVA *p*-value = 0.0006), with the highest median level found in Sawré Aboy (10.1 µg/g) and similar levels found in Poxo Muybu and Sawré Muybu (5.8 µg/g and 4.3 µg/g, respectively). Alarmingly, 93.8% of all individuals exceeded the safe limit of 2.0 µg/g, and in the village of Sawré Aboy, 100% of individuals had hair Hg higher than 2.0 µg/g (Figure 3). Then, Hg was categorized as <2.0 µg/g vs. ≥2.0 µg/g and <5.5 µg/g vs. ≥5.5 µg/g to investigate whether the sociodemographic and clinical characteristics (Supplementary Table S1) and *GSTP1* genotypes (Figure 4B,C) of the individuals were associated with Hg exposure levels. Fish consumption was associated with Hg levels, with 52 (80%) individuals with Hg ≥ 2.0 µg/g consuming fish more than twice a week (and 20% individuals with Hg < 2.0 µg/g never consuming fish) and 28 (70%) individuals with Hg ≥ 5.5 µg/g consuming fish more than twice a week (and 19.5% individuals with Hg < 5.5 µg/g never consuming fish) (Supplementary Table S1). 

Of the 82 children, only one DNA sample did not amplify for the *GSTP1* rs1695 *A>G* SNP, a successful genotyping rate of 98.8%, and the SNP was in the Hardy–Weinberg Equilibrium (HWE) for the overall population (*p*-value = 0.53). The distribution of the SNP was statistically different between the villages (*p*-value < 0.0001), with the wild genotype *GSTP1 GG* present only among the residents of Sawré Muybu (Figure 4A). No statistically significant differences were found for the *GSTP1* rs1695 SNP distributions when comparing the Hg levels (<5.5 μg/g vs. ≥5.5 μg/g or <2.0 μg/g vs. ≥2.0 μg/g) (Figure 4B,C) or the Denver II results (passed versus failed) (Figure 4D).

**Figure 5:**
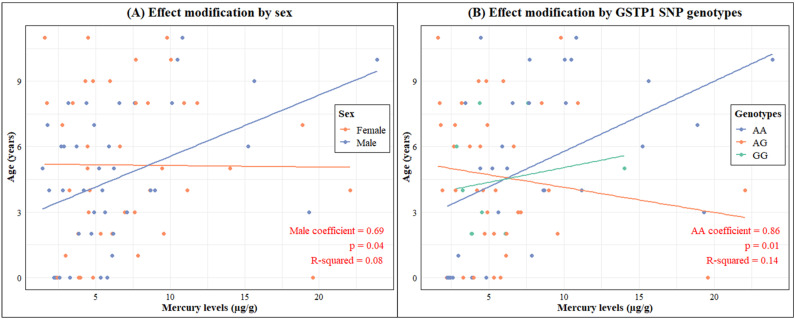
Correlation between mercury levels and age modified by sex (**A**) and the *GSTP1 313 A>G* genotypes (**B**).

Linear regression models were created to investigate the relationship between age and Hg levels, with the interaction of sex and the *GSTP1* rs1695 *A>G* SNP. It was observed that the relationship between age and Hg levels differed between the sexes, with males showing a 0.69 µg increase in Hg levels for each year increase in age (*p* = 0.04), adjusted for fish consumption and Brazilian nut consumption (Figure 5A). The *GSTP1* rs1695 SNP significantly interacted with age (estimated coefficient = 0.86, *p* = 0.01) and, as expected, showed an increase in the Hg levels in the individuals with the *GSTP1 AA* genotype, explaining approximately 14% of the variability in the Hg levels (adjusted R-squared = 0.14), adjusted for sex, fish consumption, and Brazilian nut consumption as confounder factors (Figure 5B).

Table 2 shows the sociodemographic and clinical characteristics of the nine children who failed the Denver II test and their *GSTP1* rs1695 *A>G* genotypes, together with those of their respective parents. Approximately 89% of the children had the *GSTP1* rs1695 *AA* or *AG* genotypes, which have previously been associated with high mercury levels and neurological changes in adults of the same ethnicity [15]. In addition, worryingly, 100% of the children exceeded the safe limit of 2.0 µg/g, and 55.6% (n = 5) were above the median of the total population (5.5 µg/g). The range of age was 9 months to 5 years old. Moreover, 66.7% (n = 6) were females, 33.3% (n = 3) had anemia, and 66.7% (n = 6) consumed fish between two and five times a week.

## 4. Discussion

The present work describes the interaction between age and an SNP from a gene involved in Hg metabolism on the Hg exposure levels in 82 indigenous Munduruku children from a region of the Brazilian Amazon affected by illegal mining activities. In addition, individuals carrying the *GSTP1* rs1695 *AA* genotype were found to have an increase in hair Hg levels compared to carriers of the other genotypes. Furthermore, we found that high levels of hair Hg resulting from chronic exposure, parents’ chronic exposure to Hg, impaired neurological function, the presence of the *GSTP1* rs1695 SNP, as well as poor nutritional status appear to have an influence on child neurodevelopment as assessed using the Denver II test.

The main route of exposure to MeHg for indigenous people in the Brazilian Amazon is the consumption of contaminated fish [35,36,37], but fish consumption cannot be viewed as the only villain. There are several benefits to fish consumption, such as the high amount of essential nutrients (i.e., minerals and vitamins) and polyunsaturated fatty acids (e.g., omega-3 and omega-6) [38]. Furthermore, in the context of indigenous populations in the Brazilian Amazon, sociocultural, environmental, and economic factors emphasize fish as the main source of protein in the diet [39]. In the present study, it was observed that more frequent fish consumption was associated with higher hair Hg levels; however, higher Hg exposure levels were not found in Sawré Muybu, the village with the highest fish consumption, but in Sawré Aboy, the village closest to mining activities. As described by Basta et al. (2021), proximity to mining activities should cause higher Hg contamination in fish, and therefore, higher exposure levels may be found in the residents of this region [8]. In addition, the study in [8] described the sociodemographic and clinical features of the residents of the three villages in the present study, and people who lived in Sawré Aboy had a higher prevalence of anemia, worse sanitation conditions, and lower socioeconomic status. These factors can negatively impact individuals’ health and, therefore, contribute to elevated Hg exposure levels [8,40,41].

Hg exposure levels are expected to increase with age, especially in scenarios of chronic environmental exposure [42,43,44]. It is known that variables such as sex [43,45] and genetic polymorphisms [24,46,47,48] can influence Hg exposure levels. Here, the *GSTP1* rs1695 SNP was positively correlated with Hg levels and age, with *GSTP1* rs1695 *AA* carriers showing a 0.86 µg increase in Hg levels for each year of age increase. Recently, the *GSTP1* rs1695 *AA* and *AA+AG* genotypes were associated with higher odds of having elevated Hg levels (OR = 4.2, 95%CI = 1.1–16.9 and OR= OR = 4.7, 95%CI = 1.2–17.7, respectively) in a previous study with adult Munduruku indigenous people [15]. Once in the organism, MeHg is metabolized mainly via conjugation to the small tripeptide glutathione (GSH), catalyzed by glutathione S-transferases, especially the pi 1 isoform (GSTP1), which allows its elimination in the bile [26,27]. The glutathione S-transferase pi 1 isoform enzyme is encoded by the *GSTP1* gene, which is a polymorphic member of the GST family. The *GSTP1* rs1695 *A>G* SNP alters the geometry of the substrate binding site of the enzyme by changing the amino acid at position 105 from isoleucine to valine (Ile105Val), thereby reducing substrate affinity in vitro. Therefore, it is of great importance to study SNPs in the genes involved in MeHg metabolism, such as the *GSTP1* rs1695 SNP, in populations chronically exposed to Hg [15,46,47,48,49,50,51] to identify individuals at greater risk for higher Hg levels and the subsequent development of neurocognitive disorders. 

Notably, the highest prevalence of the *GSTP1* rs1695 *AA* genotype, previously associated with higher Hg levels [15,46,47], was found among the residents of Sawré Aboy, the village with the highest internal Hg doses. In addition, the vast majority (89%) of the children who failed the Denver test carried the *GSTP1* rs1695 *AA* or *AG* genotype, and 100% of the children exceeded the safe limit of 2.0 µg/g [34]. The three children from the village of Sawré Aboy who failed the Denver test had the GSTP1 rs1695 AA genotype, two of whom were only four years old and already had very high levels of the metal in their bodies (9 and 11 μg/g). Chronic exposure to high levels of Hg combined with the genetic profile of a slow metabolizer of the metal must have contributed to the early neurotoxic effects, as one of the children was only 11 months old. These alarming data must be used to call for immediate action to end the use of Hg in this region, in addition to remedying the damage that has already occurred in terms of the health of these children. The *GSTP1* rs1695 *AG* genotype was previously associated (~4 fold) with the clinical signs of polyneuropathy in adults of the same ethnicity [15]. There is substantial evidence that high levels of Hg may be associated with neurological impairment in children [3,4,12,13] and that genetic polymorphisms can influence this association [12,25,48,51]. These findings highlight a public health concern and the need to monitor these children before they develop more serious health consequences.

Other previous works of our group comprised the adult population of these same villages [15,16], therefore, we were able to assess the data from the neurological evaluation and the *GSTP1* rs1695 *A>G* genotype of the children’s parents. It is clear how these families, chronically exposed to Hg, marginalized, and abandoned by public authorities, live in precarious conditions, with a high probability of becoming seriously ill at an early age due to the high concentrations of Hg in their bodies. Parental cognitive factors, psychosocial factors, low education, exposure to violence, and lack of early social interactions can negatively affect children’s neurodevelopment, and therefore, early interventions to reduce exposure to risk factors can reverse this outcome [19,20]. Ideally, indigenous peoples should have access to adequate health care, nutrition, education, and income as minimum conditions for development. However, the absence of these conditions, combined with mercury exposure, requires interventions to minimize harm [9]. Early intervention consists of ensuring access to health care, enabling specialized diagnoses by neurologists and psychologists, and complementary investigations to rule out other factors. This includes the notification of detected cases and drug treatment if necessary. After a diagnosis, there should be access to rehabilitation with physiotherapists, speech therapists, occupational therapists, educators, etc. In addition, nutritional advice on the safe and contamination-free consumption of fish can be provided with a view to preventing new cases. 

Nutritional status is also known to be a risk factor for impaired neurodevelopment in children [9,19], and in this regard, in our sample, cases 6, 7, and 9 had at least one nutritional alteration (being stunted and/or underweight), and in addition, two out of four children with nutritional impairments had failed more than one domain of the Denver II test. In addition, cases 6, 7, and 9 carried the *GSTP1* rs1695 *AG*, *AA*, and *AA* genotypes, respectively, which have been previously associated with high Hg levels [15]. Case 2, who also carried the *GSTP1* rs1695 *AG* genotype, was only 11 months old and already had a very high internal dose of Hg (19.6 µg/g). Although she had a normal nutritional status, her Hb level (10.7 g/dL) showed the presence of anemia, and she failed the Denver II gross motor skill test after evaluating body motor control, sitting, walking, jumping, and other movements performed by large muscles. Since the family’s fish consumption was relatively low (three times per week) compared to the other residents of this population, it is hypothesized that this child may have been exposed to mercury primarily through breastfeeding, as her mother’s hair Hg was above the reference limit (6.18 µg/g) [30] and she also had the genotype (*GSTP1* rs1695 *AG*) associated with high Hg levels [15]. There is a challenge in measuring fish consumption due to the variation between the seasons of the year and availability in the region [8]. Therefore, the information on family fish consumption may not refer to the period in which the hair samples were collected, and, besides, Hg levels reflect the moment in which the individuals were assessed and not the exposure throughout life.

Some limitations of this study must be noted, such as the fact that cross-sectional studies may not be able to assess individuals who have not yet developed the outcome. In addition, it is not possible to diagnose these children with developmental disabilities because the Denver II is a triage test, and several known risk factors for this outcome were not measured in this study. In addition, it was not possible to quantify exactly how much each observed variable contributed to the child’s neurodevelopment, but it is believed to be a multifactorial outcome influenced by exposure levels, genetic factors, nutritional status, sanitation conditions, and a diet rich in fish, among others. However, our results added to the situation of vulnerability already demonstrated in this population. For example, the studies in [8,11,14,16,30,36] are indicative that these children need to be monitored for the application of more robust tests that allow definitive diagnoses for subsequent referrals and the implementation of effective public policies.

## 5. Conclusions

This is the first study to evaluate the effect of the *GSTP1* rs1695 A>G polymorphism on Hg exposure and neurodevelopment in indigenous children from the Brazilian Amazon. The residents of Sawré Aboy had significantly higher Hg levels, as well as a higher prevalence of the *GSTP1* rs1695 *AA* genotype, previously associated with higher Hg levels in the adult population of the same villages. Male children and *GSTP1* rs1695 *AA* carriers tended to have increased Hg levels over the years. All nine children with neurodevelopmental disorders had Hg levels above the safe limit, and eight of them had the *GSTP1* rs1695 *AA* or *AG* genotypes. Our results highlight the vulnerable situation in which these children live, given the presence of the early symptoms of Hg contamination and the role of genetic alterations in this process. In conclusion, there is a clear need to monitor this population to provide specific treatment. Therefore, the genetic counseling of this population is important to identify the individuals more susceptible to Hg accumulation and, consequently, at higher risk of neurodevelopmental impairment.

## Figures and Tables

**Table 1:** Sociodemographic and clinical characteristics of the study population, according to the village of residence in the Sawré Muybu indigenous territory, Pará State, Brazilian Amazon, in 2019 (n = 82).

Characteristics	Overall (n = 82)	Village			*p*-Value ^a^
		Poxo Muybu	Sawré Aboy	Sawré Muybu	
		(n = 28)	(n = 15)	(n = 39)	
**Sex**					
Female	42 (51.2)	16 (57.1)	5 (33.3)	21 (53.8)	0.30
Male	40 (48.8)	12 (42.9)	10 (66.7)	18 (46.2)	
**Age (years)**					
0–4	48 (58.5)	16 (57.1)	9 (60.0)	23 (59.0)	0.98
≥5	34 (41.5)	12 (42.9)	6 (40.0)	16 (41.0)	
**Height for age ^b,c^**					
Severely stunted	3 (4.1)	1 (4.3)	0 (0.0)	2 (5.3)	0.82
Moderately stunted	14 (19.2)	3 (13.0)	3 (25.0)	8 (21.1)	
Normal	56 (76.7)	19 (82.6)	9 (75.0)	28 (73.7)	
**Weight for age ^b^**					
Severely underweight	1 (1.4)	0 (0.0)	0 (0.0)	1 (2.6)	0.80
Moderately underweight	6 (8.1)	2 (8.3)	1 (8.3)	3 (7.9)	
Normal	66 (89.2)	21 (87.5)	11 (91.7)	34 (89.5)	
Overweight	1 (1.4)	1 (4.2)	0 (0.0)	0 (0.0)	
**Weight for height ^d^**					
Eutrophy	35 (83.3)	8 (72.7)	9 (100.0)	18 (81.8)	0.26
Overweight risk	7 (16.7)	3 (27.3)	0 (0.0)	4 (18.2)	
**BMI for age ^e^**					
Eutrophy	35 (89.7)	13 (81.2)	6 (100.0)	16 (94.1)	0.32
Overweight risk	4 (10.3)	3 (18.8)	0 (0.0)	1 (5.9)	
**Anemia ^f^**					
No	63 (80.8)	22 (81.5)	9 (69.2)	32 (84.2)	0.49
Yes	15 (19.2)	5 (18.5)	4 (30.8)	6 (15.8)	
**Fish consumption**					
None	9 (11.0)	3 (10.7)	2 (13.3)	4 (10.3)	0.002
≤2 times a week	20 (24.4)	14 (50.0)	3 (20.0)	4 (7.7)	
>2 times a week	53 (64.6)	11 (39.3)	10 (66.7)	32 (82.1)	
**Nut consumption**					
None	13 (15.9)	4 (14.3)	2 (13.3)	7 (17.9)	0.12
Daily	37 (45.1)	11 (39.3)	8 (53.3)	18 (46.2)	
Weekly	18 (22.0)	5 (17.9)	1 (6.7)	12 (30.8)	
Monthly	14 (17.1)	8 (28.6)	4 (26.7)	2 (5.1)	
**Denver II result ^g^**					
Passed	46 (83.6)	18 (94.7)	7 (70.0)	21 (80.8)	0.20
Failed	9 (16.4)	1 (5.3)	3 (30.0)	5 (19.2)	

^a^ *p*-value obtained from the Chi-squared test (Pearson’s *p*-value) or Fisher’s exact test when needed. ^b^ Z-scores obtained for the measures of height and weight for age for children under 0 to 10 years old (n = 74). ^c^ Missing information from one individual (n = 73). ^d^ Z-scores obtained for the measures of weight for height for children under 5 years old (n = 42). ^e^ Z-score obtained for the measure of BMI for age for children older than 5 years and missing information from one individual (n = 39). ^f^ Missing information from four individuals. ^g^ Results from the Denver II developmental screening test for children from 0 to 6 years old (n = 55).

**Table 2:** Sociodemographic and clinical characteristics of the children who failed the Denver II developmental screening test in the Sawré Muybu indigenous territory, Pará State, Brazilian Amazon, in 2019 (n = 9).

Case	Village ^a^	Age	Sex	Nutritional Status ^b^	Anemia	Fish Consumption	Denver II Failure ^c^	Hg ^d^	*GSTP1 A>G* ^e^	Parent’s Neurological Impairment ^f^	Parent’s *GSTP1A>G* ^g^
1 ^h^	SM	9 months	F	Overweight risk	No	3 times/day	Language	2.4	***AA***	Yes	*AG*/*AG*
2	SM	11 months	F	Normal	Yes	3 times/week	Gross motor	**19.6**	*AG*	Yes	*AG*/*AG*
3 ^i^	SA	11 months	M	Normal	Yes	2 times/week	Language	2.6	***AA***	Yes	***AA***/*AG*
4 ^h^	SM	2 years	F	Normal	No	3 times/day	Language	3.8	*GG*	Yes	*AG*/*AG*
5	SM	3 years	F	Normal	No	3 times/week	Language	**6.9**	*AG*	Yes	*AG*/*AG*
6	SM	4 years	F	Moderately stunted	No	3 times/day	Language and fine motor	4.6	*AG*	Yes	*AG*/*GG*
7	SA	4 years	F	Moderately stunted and moderately underweight	Yes	5 times/week	Language	**11.2**	***AA***	Yes	*AG*/***AA***
8 ^i^	SA	4 years	M	Normal	No	2 times/week	Language	**8.7**	***AA***	Yes	***AA***/*AG*
9	PM	5 years	M	Moderately underweight	No	3 times/week	All domains	**6.2**	***AA***	Yes	***AA***/MI ^j^

^a^ Sawré Muybu (SM), Sawré Aboy (SA), and Poxo *Muybu* (PM). ^b^ Nutritional status according to the Z-scores, as shown in Table 1. ^c^ Domains failed during the Denver II test. ^d^ Internal dose of mercury presented as µg/g. ^e^ Genotypes *GSTP1* rs1695 *AA* and *AG* were previously associated with elevated Hg levels and neurological impairment, respectively, among adults from the same population [15]. ^f^ All cases had at least one parent with neurological impairment (somatosensory, motor, or cognitive). ^g^ Parents’ genotypes for the *GSTP1* rs1695 *A>G* polymorphism (mother’s genotype/father’s genotype). ^h^ Cases 1 and 4 were sisters. ^i^ Cases 3 and 8 were siblings. ^j^ MI = missing information. Additional information: these nine children never had malaria in their lives.

## Data Availability

Data are contained within the article and Supplementary Materials.

## Supplementary Materials

The following supporting information can be downloaded at: https://www.mdpi.com/article/10.3390/toxics12060441/s1, Table S1: Distribution of demographic and clinical characteristics according to the Hg internal dose (n = 81).
