# Supplementary material for: The GSTP1 rs1695 Polymorphism Is Associated with Mercury Levels and Neurodevelopmental Delay in Indigenous Munduruku Children from the Brazilian Amazon
Source: Toxics. 2024 Jun 19;12(6):441. doi: 10.3390/toxics12060441 (PMC11209255; doi:10.3390/toxics12060441)
Supplement: Supplementary file 1 [file toxics-12-00441-s001.zip › toxics-3054594-supplementary.pdf]

Table S1. Distribution of demographic and clinical characteristics according to the Hg internal dose (n=81).

Characteristics	Hg levels (µg/g)[a]			Hg levels (µg/g)[a]		
	< 2.0 (n= 5)	≥ 2.0 (n= 76)	P-value[b]	< 5.5 (n= 41)	≥ 5.5 (n= 40)	P-value[b]
**Sex**						
Female	2 (40.0)	39 (51.3)	0.98	19 (46.3)	22 (55.0)	0.58
Male	3 (60.0)	37 (48.7)		22 (53.7)	18 (45.0)	
**Age**						
≤ 5	2 (40.0)	45 (59.2)	0.71	26 (63.4)	21 (52.5)	0.44
> 5	3 (60.0)	31 (40.8)		15 (36.6)	19 (47.5)	
**Denver II Result**						
Passed	2 (100.0)	43 (82.7)	0.99	26 (86.7)	19 (79.2)	0.71
Failed	0 (0.0)	9 (17.3)		4 (13.3)	5 (20.8)	
**Weight for Age**[c]						
Severe underweight	0 (0.0)	1 (1.4)		1 (2.6)	0 (0.0)	
Moderate underweight	1 (25.0)	5 (7.2)	0.65	3 (7.7)	3 (8.8)	0.56
Normal	3 (75.0)	62 (89.9		35 (89.7)	30 (88.2)	
Overweight	0 (0.0)	1 (1.4)		0 (0.0)	1 (2.9)	
**Height for Age**[c]						
Severely stunted	0 (0.0)	3 (4.4)		3 (7.7)	0 (0.0)	
Moderately stunted	1 (25.0)	12 (17.6)	0.86	6 (15.4)	7 (21.2)	0.24
Normal	3 (75.0)	53 (77.9)		30 (76.9)	26 (78.8)	
**BMI for Age**[d]						
Eutrophy	4 (100.0)	31 (88.6)	0.99	16 (88.9)	19 (90.5)	0.99
Overweight risk	0 (0.0)	4 (11.4)		2 (11.1)	2 (9.5)	
**Weight for Height**[e]						
Eutrophy	1 (100.0)	33 (82.5)	0.99	18 (78.3)	16 (88.9)	0.63
Overweight risk	0 (0.0)	7 (17.5)		5 (21.7)	2 (11.1)	
**Anemia**						
No	5 (100.0)	58 (80.6)	0.62	33 (89.2)	30 (75.0)	0.19
Yes	0 (0.0)	14 (19.4)		4 (10.8)	10 (25.0)	
**Fish consumption**						
None	2 (40.0)	6 (7.9)		8 (19.5)	0 (0.0)	
≤ 2 times a week	2 (40.0)	18 (23.7)	**0.03**	8 (19.5)	12 (30.0)	**0.01**
> 2 times a week	1 (20.0)	52 (68.4)		25 (61.5)	28 (70.0)	

**Nut consumption**						
None	0 (0.0)	12 (15.8)		10 (24.4)	2 (5.0)	
Daily	2 (40.0)	35 (46.1)	0.16	18 (43.9)	19 (47.5)	0.05
Weekly	3 (60.0)	15 (19.7)		9 (22.0)	9 (22.5)	
Monthly	0 (0.0)	14 (18.4)		4 (9.8)	10 (25.0)	

[a]Internal dose of mercury presented as µg/g. [b]p-value obtained from the Chi-squared Test (Pearson p-value) or Fisher's exact test, when needed. [c]Z-scores obtained for the measures of height and weight for age for those children under from 0 to 10 years old. [d]Z-scores obtained for the measures of weight for height, for those children under 5 years old. [e]Z-score obtained for the measure of BMI for age, for those children older than 5 years.
